# Biological Sunglasses in a Deep-Sea Squid: Pigment Migration in the Retina of *Gonatus onyx*

**DOI:** 10.3390/vision8020026

**Published:** 2024-04-25

**Authors:** Ryan B. Howard, Jessica Kniller, Kathrin S. R. Bolstad, Monica L. Acosta

**Affiliations:** 1 AUT Lab for Cephalopod Ecology & Systematics, School of Science, Auckland University of Technology, Auckland 1010, New Zealand; ryan.howard@aut.ac.nz (R.B.H.);; 2 School of Optometry and Vision Science, New Zealand National Eye Centre (NZ-NEC), University of Auckland, Auckland 1010, New Zealand; 3 Centre for Brain Research (CBR), University of Auckland, Auckland 1010, New Zealand

**Keywords:** cephalopod, squid, pigment migration, ommin, neurotransmitters, retina, vision

## Abstract

The outward migration of ommin pigment granules from the bases to the tips of the photoreceptors in response to light has been reported in the retina of several (mostly coastal) squid species. Following exposure to light and then dark conditions, we collected and processed retinal tissue from juvenile specimens of a deep-sea oegopsid squid, *Gonatus onyx*. We aimed to determine whether the ommin pigment returns to baseline, and to investigate the presence of glutamate neurotransmitter signaling under both dark and light conditions. We confirmed the presence of ommin granules but observed variability in the return of pigment to the basal layer in dark conditions, as well as changes in glutamate distribution. These findings provide support for the migration of retinal ommin pigment granules as a mechanism for regulating incoming light.

## 1. Introduction

Eye evolution in animals that are primarily active under dark conditions has followed several strategies. The ability to moderate the intensity of light entering the eye may be particularly important for species exposed to a wide range of light conditions, such as shallow-dwelling organisms exposed to regular day/night cycles, or species that inhabit diverse oceanic layers across their lifespan. One physiological response to changing light conditions, reported in several cephalopods to date, is the migration of ommin pigment granules within the retina, providing a visual screening effect akin to ‘biological sunglasses’.

In cephalopods, evidence of ocular screening pigments has existed since the late 19th century [1]. In 1959, Butenandt [2] identified the ommochrome pigmentation as ommin in the retinas of two squids (*Todarodes sagittatus* and *Alloteuthis subulata*), the European common cuttlefish (*Sepia officinalis*) and two octopuses (*Octopus vulgaris* and *Eledone moschata*). Daw and Pearlman [3] showed that in the coastal market squid *Doryteuthis pealeii*, ommin granules could migrate to the distal end of the of the outer retinal layer, providing a filter equivalent to 0.6 log units. This outward migration had a spectral sensitivity equivalent to rhodopsin and occurred in 5–15 min, once 3–10% of the rhodopsin had isomerized. After the light stimulus was removed, the pigment granules returned to their original basal position over a further 5–15 min. However, if more than 10% of the rhodopsin was isomerized, the screening pigment instead took several hours to fully migrate inward under restored dark conditions. Notably, pigment migration occurred only when light fell directly on the rhabdomeres, and not during the illumination of the granules themselves [4]. These observations supported the prevailing hypothesis that outward ommin migration occurs during bright ambient light conditions to block intense light and prevent photoreceptor oversaturation, with a quick return to the ommin layer upon the return to darkness.

In cephalopods, screening pigments have mostly been found in better-studied nearshore and shallow-water species, which experience changing light conditions due to daylight cycles [3,5,6,7]. However, screening pigment granules were also recently recognized in the oegopsid (deep-sea) ‘glass’ squid *Teuthowenia pellucida*, and their position within the retina suggested different stages of light/dark adaptation at the time of specimen preservation [8]. In deep-sea squids, migratory pigment granules could provide an advantage during diel vertical migrations, or during descent into deeper layers as they mature. The mechanisms governing pigment migration during dark adaptation are different from the mechanisms controlling migration during light adaptation [9] and may even differ among species of the same group.

In the present paper, we investigated the timing of ommin pigment migration under dark conditions, and whether glutamate neurotransmitter levels change during dark adaptation, which is believed to be under the control of dopaminergic efferent innervation regulating the retraction of ommin. Enzymes that synthesize glutamate, an excitatory neurotransmitter (the same neurotransmitter used by vertebrate photoreceptors), have been found within the photoreceptors of the hummingbird ‘bobtail squid’, *Euprymna berryi* [10]. Additionally, glutamate levels in the retina of *Sepia officinalis* can be influenced by light/dark environments [11].

This study investigates retinal screening pigment migration in a pelagic oegopsid squid species primarily found throughout the North Pacific. *Gonatus onyx*, known as the ‘black-eyed’ squid, is the most encountered gonatid near California, and is frequently observed at depths of 375–925 m (maximum 1975 m) by remotely operated vehicles operating in the Monterey Canyon [12]. Like many deep-sea oegopsids, *G. onyx* inhabits deeper oceanic strata as it matures [13]; females have been observed brooding egg masses at depths of 1250–2500 m [13,14]. This species therefore experiences a range of light conditions across its lifespan (and potentially across shorter timeframes), prompting us to investigate the possibility of screening pigmentation migration in a cohort of juveniles under a controlled series of light/dark exposures.

## 2. Materials and Methods

### 2.1. Specimen Acquisition

**Figure 1:**
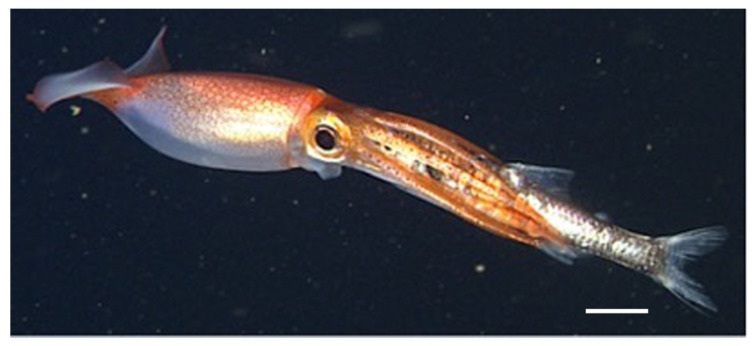
Juvenile ‘black-eyed’ squid, *Gonatus onyx*, feeding on a myctophid. © Monterey Bay Aquarium Research Institute. Scale bar = 1 cm.

Live juvenile gonatid squid (*Gonatus onyx*, ML 10–20 mm; Figure 1) were trawled after sundown at a depth of 350 m by the Monterey Bay Aquarium Research Institute (MBARI) aboard the RV *Western Flyer* over the Monterey Canyon (36°41.4–42.8′ N, 122°10.3–13.3′ W for individuals G01, G12–G13; 36°32.3–35.4′ N, 122°31.8–35.3′ W for G02–G11) in March 2013. Thirteen individuals were used in this study.

### 2.2. Experimental Design

After net retrieval, the 13 juveniles were sorted while exposed to ambient light under bright fluorescent bulbs (F24T12 35W linear fluorescent tube, 4200 k, 380–760 nm, OSRAM Sylvania, Wilmington, NC, USA) with an illuminance of 350 lux measured with an LX-103 Digital Light Meter (Lutron Electronic Enterprise, Taipei City, Tawain). During experiments, each specimen was sorted from the trawl catch and placed into a small container of seawater, in which it was exposed to light for a total (including sorting time) of 20, 30 or 90 min, and subsequently moved into a lightless room for variable periods of time. Of the 13 juvenile *G. onyx* processed under light, 11 specimens were selected to assess the migration and return of the pigment granules within the retina after subsequent dark adaptation. The remaining two specimens were used to identify the presence of the neurotransmitter glutamate in the retina (Table 1).

### 2.3. Pigment Migration

Five individuals were exposed to 350 lux of fluorescent lighting for 20 min (G02–G06, Group 1), while five individuals were exposed to the same light for 30 min (G07–G11, Group 2). The light exposure paradigm was determined by the time required to sort the catch. The 10 min difference in processing time provided an opportunity to compare pigment migration differences; an extra 10 min light exposure could have a significant impact according to a previously reported dark/light adaptation study [3]. After light exposure, specimens were then placed into individual containers within a darkened cold room (<60 lux), with one individual euthanized at 15 min and every 15 min interval thereafter until 75 min (Group 1) or after 20 min and every 10 min thereafter until 60 min (Group 2). Two specimens appeared moribund at the time of euthanasia and were subsequently removed from the results (Group 1, 30 min dark adaptation, G03; Group 2, 40 min dark adaptation, G09).

Specimens were immediately fixed whole after euthanasia in 5% buffered formalin to preserve the position of ommin pigment granules. Later, a single eye from each fixed specimen was removed and fixed again in 4% formaldehyde in phosphate-buffered solution (PBS) for 30 min, rinsed in PBS and stored until sectioning. The contralateral eye was fixed in glutaraldehyde. Unfortunately, glutaraldehyde fixation did not preserve the ommin pigment well, and these samples were not processed further.

One specimen (G01, ML 30 mm) served as the negative control for pigment migration analysis; this individual was exposed to 90 min of light at 350 lux, then subjected to darkness for 24 h, then euthanized. However, the PFA-fixed eye was damaged beyond use and only the contralateral eye preserved in 2.5% glutaraldehyde was used, which resulted in the poor preservation of the ommin pigment granules, although some pigment granules survived in the negative control.

Prior to sectioning, all formaldehyde-fixed tissues for pigment migration analysis were immersed in 30% sucrose solution for at least 24 h before embedding them in optimum cutting temperature medium and sectioning at 12 µm thickness using a cryostat (Leica CM3050 S, Wetzlar, Germany). Dorsal, central and ventral sections were obtained from the specimen adapted to the dark for the longest duration (G06, 20 min light/75 min dark) and from the specimen with the most exposure to light and shortest duration of dark adaptation (G07, 30 min light/20 min dark). For comparison among individuals in a group, vertical sections were obtained from the retina’s central region. All samples were stained with toluidine blue.

### 2.4. Neurotransmitter Expression

To determine whether changes in neurotransmitter presence are discernable between different dark adaptation paradigms (i.e., less than a day of dark adaptation), two juvenile squids (*G. onyx*, G12 and G13, ML 15 mm) were exposed to 30 min of light at 350 lux. G13 was immediately euthanized after light exposure with no subsequent dark adaptation. After light exposure, G12 was placed in a darkened container for 90 min to allow for partial or full dark adaptation of the retina. The eyes were then preserved in conditions identical to those for specimens analyzed for pigment migration. Samples were placed in 2.5% glutaraldehyde with 1% formaldehyde in PBS for 1 h and dehydrated in an ethanol series of increasing concentration. Fixation in glutaraldehyde rendered most of the ommin pigment granules unperceivable. These glutaraldehyde-fixed samples were then processed by epoxy resin (EPON) embedding following a standard protocol [15,16] and placed in a 3:1 resin–acetone mixture overnight. Next, the tissues were submerged in 100% EPON and placed in an oven at 60 °C for at least 24 h to polymerize the resin. The resin-embedded samples were cut into 250–500 nm thick vertical sections using a glass knife (made with a Leica EM KMR2, Wetzlar, Germany) and an ultramicrotome (Leica Ultracut UCT, Wetzlar, Germany). Sections were placed on Teflon-coated slides and processed for immunogold labeling of glutamate.

The retinas were prepared for the identification of neurotransmitters following the silver-intensified immunogold labeling procedure described by Marc et al. [15,17]. Single consecutive 250 nm sections were collected in separate wells and labeled with antibodies. The primary antibody, rabbit anti-glutamate (1:1000, ab9440, Abcam, Cambridge, UK), was used. Controls for each treatment were produced by taking retinal sections and processing them under the same conditions as the experimental samples, excluding the primary antibodies. This allowed for the assessment of any non-specific staining or background signals present without specific antibody binding. A 1.4 nm Nanogold^®^ conjugate goat anti-rabbit secondary antibody (1:100, #2003; Nanoprobes, New York, NY, USA) was used to detect the primary antibody in a silver-intensified reaction procedure [17,18]. 

### 2.5. Imaging

All images were captured with a Leica CTR MIC microscope (Wetzlar, Germany) and a Leica DC 500 camera (Wetzlar, Germany) using either 10×, 20× or 40× objectives. Images were acquired using the same parameters of exposure, contrast and brightness.

### 2.6. Barcoding

Small tissue snips were taken from the mantle of each specimen and stored separately in 95% ethanol. Due to the presence of multiple *Gonatus* species in this region, DNA barcoding (cytochrome c oxidase subunit 1 [CO1]) was used to confirm this material as *G. onyx (*>99% match to other *G. onyx* material from the same region, using the Barcode of Life Database [BOLD]; [19]). DNA was extracted and prepared at the AUT Lab for Cephalopod Ecology & Systematics (ALCES) following the protocols outlined by Braid and Bolstad [20], then sequenced at Macrogen, Korea.

## 3. Results

### 3.1. Pigment Migration

**Figure 2:**
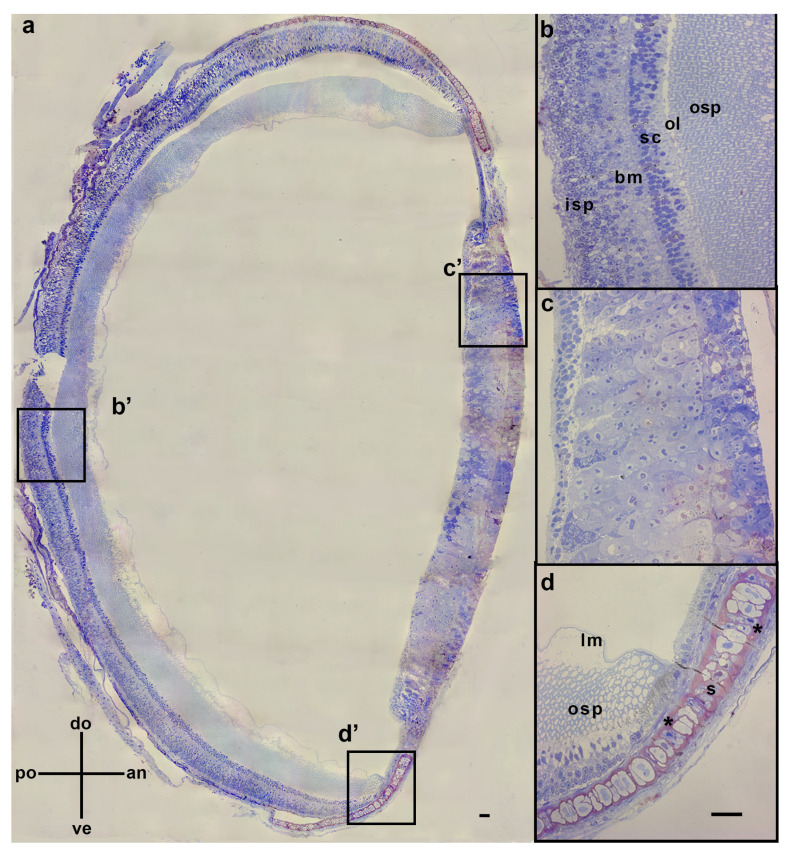
(**a**) Transverse cross-section of juvenile *G. onyx* (15 mm ML) eye. (**b**) Enlarged section of b’ depicting retinal layers. (**c**) Enlarged section of c’ depicting the iris. (**d**) Enlarged section of d’ depicting the transition from the retina to the ciliary body. The asterisks indicate the cartilaginous parts of the sclera (s). Ommin granules are not visible due to glutaraldehyde preservation (see Section 2. Abbreviations: dorsal (do), ventral (ve), posterior (po), anterior (an), inner segment of photoreceptors (isp), basal membrane (bm), supporting cells (sc), expected location of ommin layer (ol), outer segment of photoreceptors (osp), limiting membrane (lm), sclera (s). Scale bars = 20 µm.

The transverse cross-sections of the juvenile *G. onyx* eyes revealed a typical cephalopod eye structure (Figure 2), consisting of an everted retina with the distal ends of the outer segment of the photoreceptors directed anteriorly (Figure 2a). A basal membrane and supporting cells separated the outer and inner segments of the photoreceptor cells, while a limiting membrane separated the outer segment of the photoreceptors from the vitreous body (Figure 2b). In the dark-adapted eye, the ommin layer would be expected to be concentrated at the base of the outer segment of the photoreceptors. In the ventral and dorsal anterior sections of the eye, the retina was supported by a cartilaginous sclera (Figure 2d), which continued anteriorly to the body of the iris (Figure 2c).

**Figure 3:**
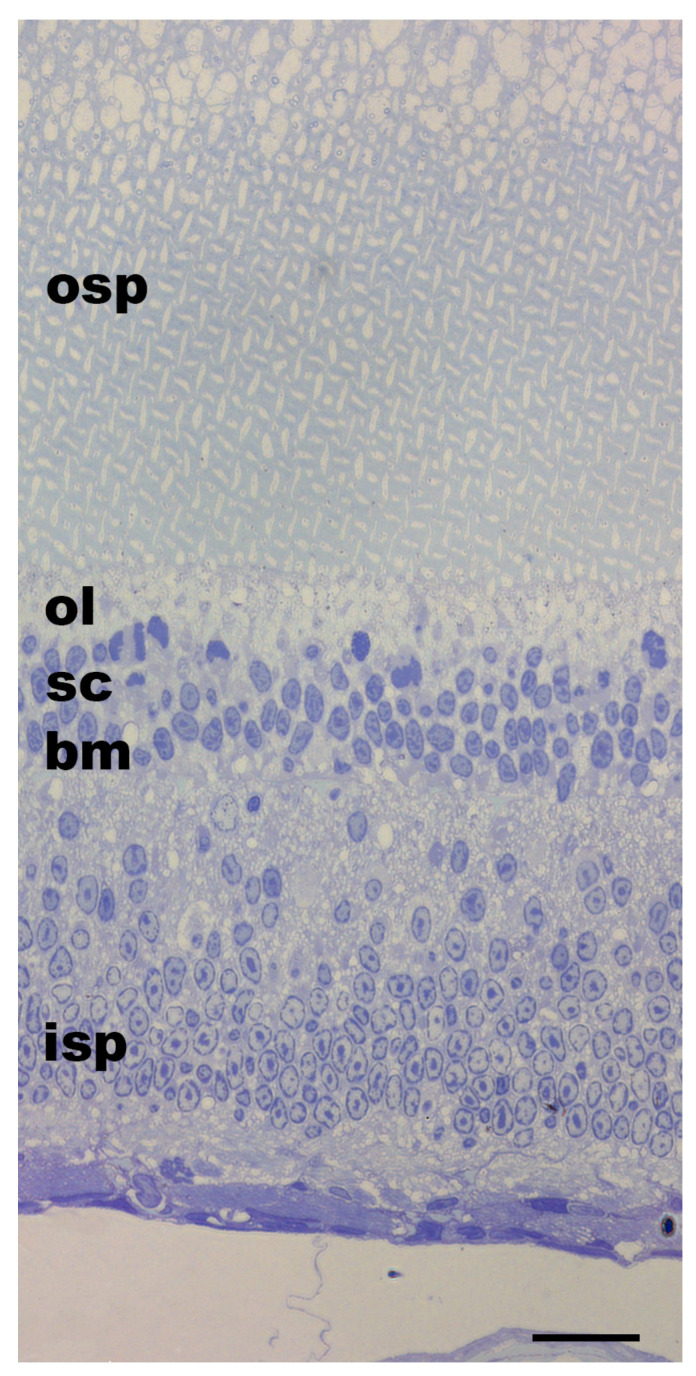
Toluidine blue staining of a horizontally sectioned juvenile *Gonatus onyx* retina at the photoreceptor level. The lattice-like arrangement of rhabdomeres in the outer segment is typical of cephalopods. Ommin granules are not visible due to glutaraldehyde preservation (see Section 2), but the ommin layer (ol) should be near the base of the outer segment of the photoreceptors (osp). Abbreviations: inner segment of the photoreceptors (isp), basal membrane (bm), supporting cells (sc), expected location of ommin layer (ol), outer segment of the photoreceptors (osp). Scale bar = 20 µm.

**Figure 4:**
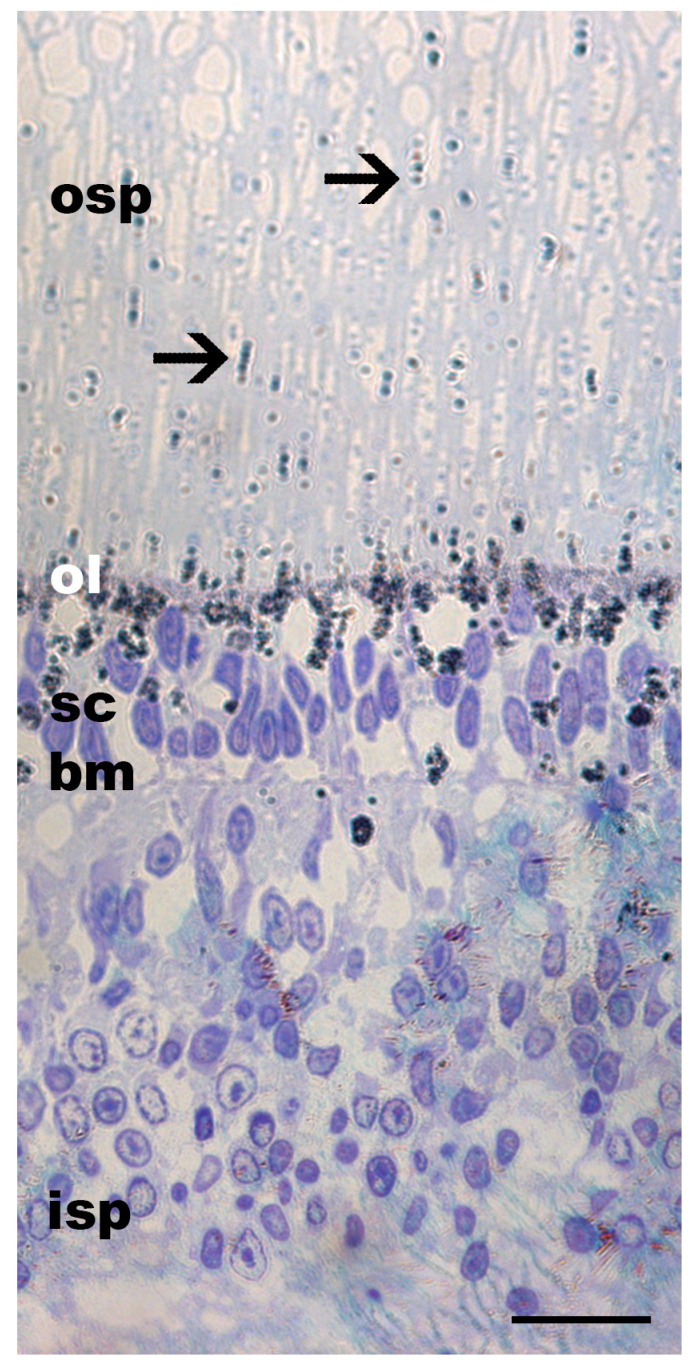
Ommin pigment location in the retina of a juvenile *G. onyx* exposed to 90 min of light at 350 lux then subjected to darkness for 24 h. The screening pigment of this specimen was preserved, revealing the single-cell resolution and location of ommin pigment granules. No pigment was observed at the distal tip of the photoreceptors. Arrows indicate location of migrating ommin pigment. Abbreviations: inner segment of the photoreceptors (isp), basal membrane (bm), supporting cells (sc), ommin layer (ol), outer segment of the photoreceptors (osp). Scale bar = 20 µm.

The photoreceptors were arranged in groups of four, forming rhabdomeres, appearing as a lattice arrangement (Figure 3). In the negative control specimen (G01), exposed to 90 min light followed by 24 h in darkness, the ommin pigment granules were dispersed throughout the outer photoreceptor layer, with most of the pigment granules found near the base of the ommin layer and in the layer of supporting cells (Figure 4).

From specimens G06 (20 min light/75 min dark) and G07 (30 min light/20 min dark), the dorsal, central and ventral horizontal sections were obtained to determine whether the return of pigment to the ommin baseline was uniform across the retinal regions.

**Figure 5:**
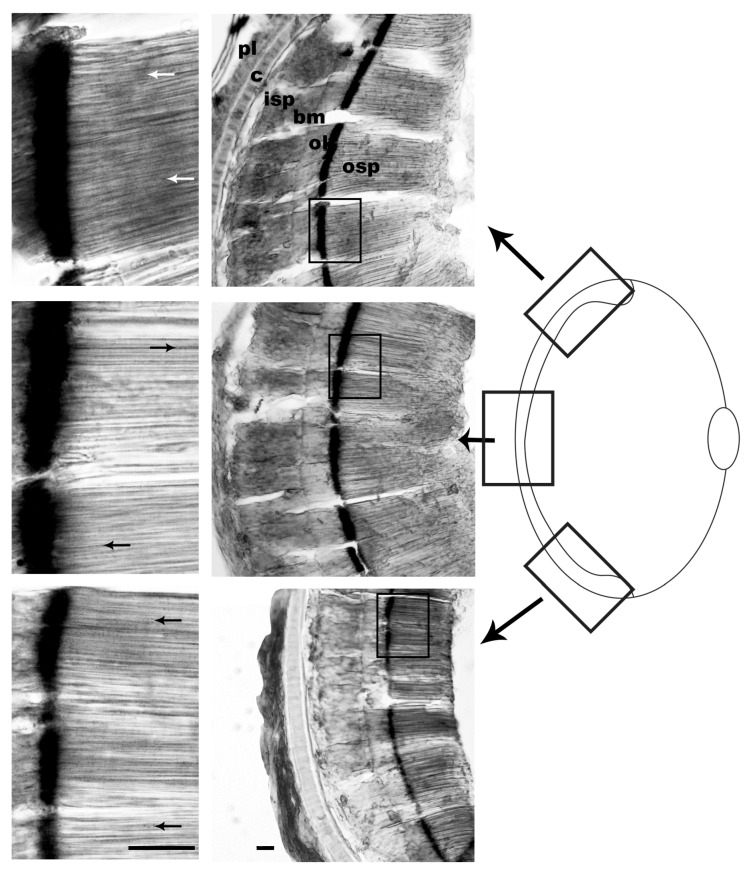
Ommin pigment location in the specimen exposed to 30 min of light at 350 lux then subjected to darkness for 20 min (G07). The screening pigment of this specimen was preserved, revealing the location of ommin pigment granules close to the basal ommin layer and absent from the tip of the photoreceptors for all retinal sections. The top row of images depicts the dorsal section of the retina, the middle row depicts the central section and the bottom row depicts the ventral section. Arrows indicate location of migrating ommin pigment. Abbreviations: outer segment of the photoreceptors (osp), ommin layer (ol), basal membrane (bm), inner segment of the photoreceptors (isp), rudimentary cartilage (c), plexiform layer (pl). Scale bars = 20 µm.

The specimen exposed to the longest light and shortest dark treatments (G07), showed scattered ommin granules distributed along the photoreceptors after 20 min of dark adaptation (Figure 5). However, all regions of the retina showed similar stages of pigment return, as nearly all the ommin granules had returned to the baseline.

**Figure 6:**
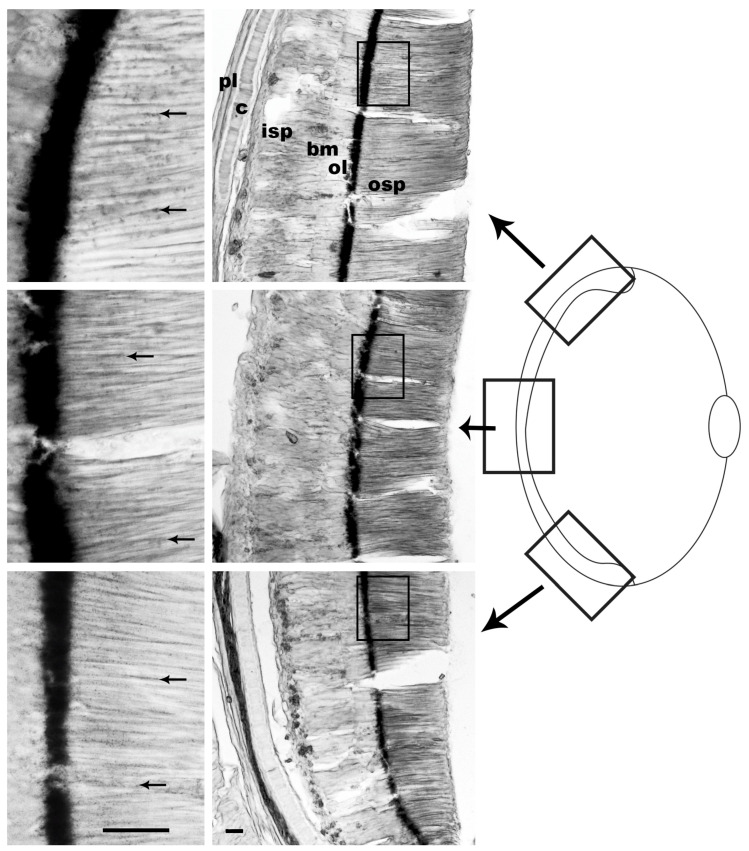
Ommin pigment location in the *G. onyx* specimen exposed to 20 min of light at 350 lux then subjected to darkness for 75 min. The screening pigment of this specimen was preserved, revealing the location of ommin pigment granules close to the ommin layer and absent from the tip of the photoreceptors. The top row of images depicts the dorsal section of the retina, the middle row depicts the central section and the bottom row depicts the ventral section. Arrows indicate location of migrating ommin pigment. Abbreviations: outer segment of the photoreceptors (osp), ommin layer (ol), basal membrane (bm), inner segment of the photoreceptors (isp), rudimentary cartilage (c), plexiform layer (pl). Scale bars = 20 µm.

In the two specimens with the longest dark adaptation periods (G06, 75 min, Figure 6; and the negative control, G01, 24 h, Figure 4), most of the ommin granules appeared to return to the basal layer, and only a few remained distributed along the photoreceptor length based on the percent length of migration (more in G06 than in G01). The visual observations for each segment of G06 showed that this trend was consistent throughout all the retinal regions.

In the two individuals where the dorsal, central and ventral retinal sections were analyzed (G06, G07), the proportions of ommin granules in the basal layer and along the photoreceptor length appeared to be consistent within each eye, regardless of their location.

**Figure 7:**
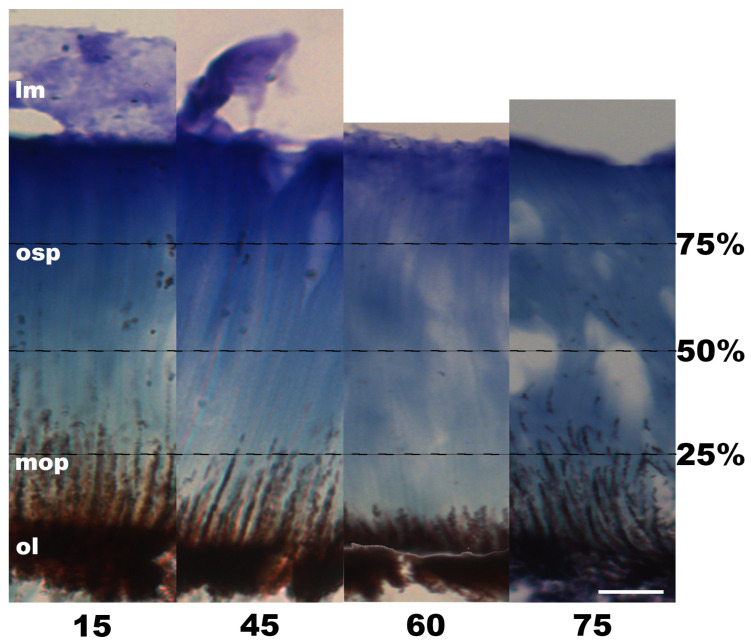
Ommin pigment granule migration in the outer segment of the photoreceptors of Group 1 juvenile (ML 10–20 mm) *G. onyx* retinas following exposure to light (350 lux) for 20 min, followed by dark (minutes indicated below each cross-section). Percentage values to the right of the image and dotted lines refer to the outer segment of the photoreceptors. Abbreviations: ommin layer (ol), migrating ommin pigment (mop), ommin-free outer segment of the photoreceptors (osp), limiting membrane (lm). All images depicted at 40× magnification. Scale bar = 20 µm.

**Figure 8:**
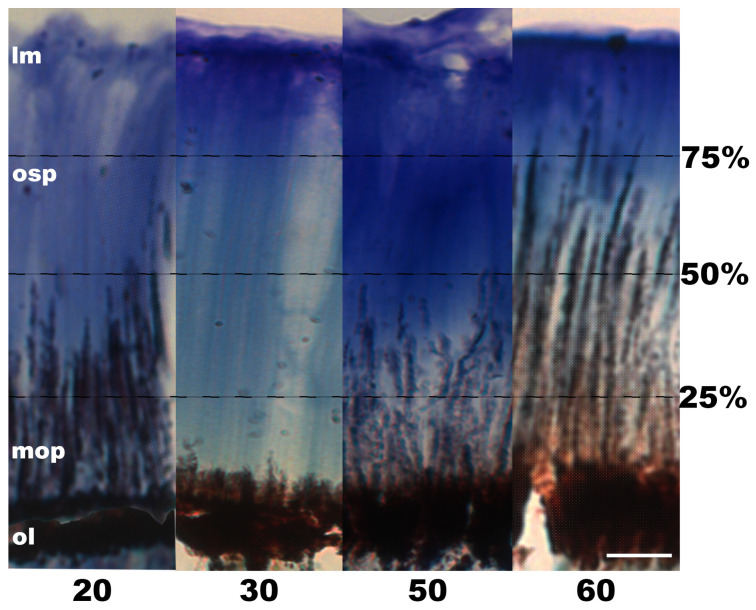
Ommin pigment granule migration in the outer segment of the photoreceptors of Group 2 juvenile (ML 10–20 mm) *G. onyx* retinas following exposure to light (350 lux) for 30 min, followed by dark (minutes indicated below each cross-section). Percentage values on the side of the image and dotted line refer to photoreceptor length. Abbreviations: ommin layer (ol), migrating ommin pigment (mop), ommin-free outer segment of the photoreceptors (osp), limiting membrane (lm). All images depicted at 40× magnification. Scale bar = 20 µm.

In the central retinal section of all the experimental samples (G02–G11), a substantial proportion of the ommin pigment granules was concentrated within the basal layer of the photoreceptors (Group 1, Figure 7; Group 2, Figure 8). In all the samples, some granules were also distributed along the photoreceptors’ length, with some variation observed among individuals.

In Group 1 (individuals exposed to 20 min of light and then periods of darkness increasing by 15 min; Figure 7), similar results were observed among the individuals returned to darkness for 15, 45 and 75 min, with most of the pigment evident in the basal 25% of the photoreceptor length. In the individual that was subjected to darkness for 60 min after light exposure (G05), the returning granules were more concentrated and positioned closer to the ommin layer, within the basal 10% of the photoreceptor length.

In Group 2 (individuals exposed to 30 min of light and then periods of darkness increasing by 10 min; Figure 8), most of the specimens showed widely distributed ommin pigment granules in the photoreceptors. The individuals that were returned to darkness for 20 and 50 min post light exposure (G07 and G10, respectively) exhibited granules across the basal 40–50% of the photoreceptor length; in the 60 min dark-exposed individual (G11), the migrated granules spanned the majority (basal ~80%) of the photoreceptor length. In the individual exposed to 30 min of darkness (G08), the granules were more concentrated near the ommin layer, within the basal 10% of the photoreceptor length.

### 3.2. Neurotransmitter Expression

**Figure 9:**
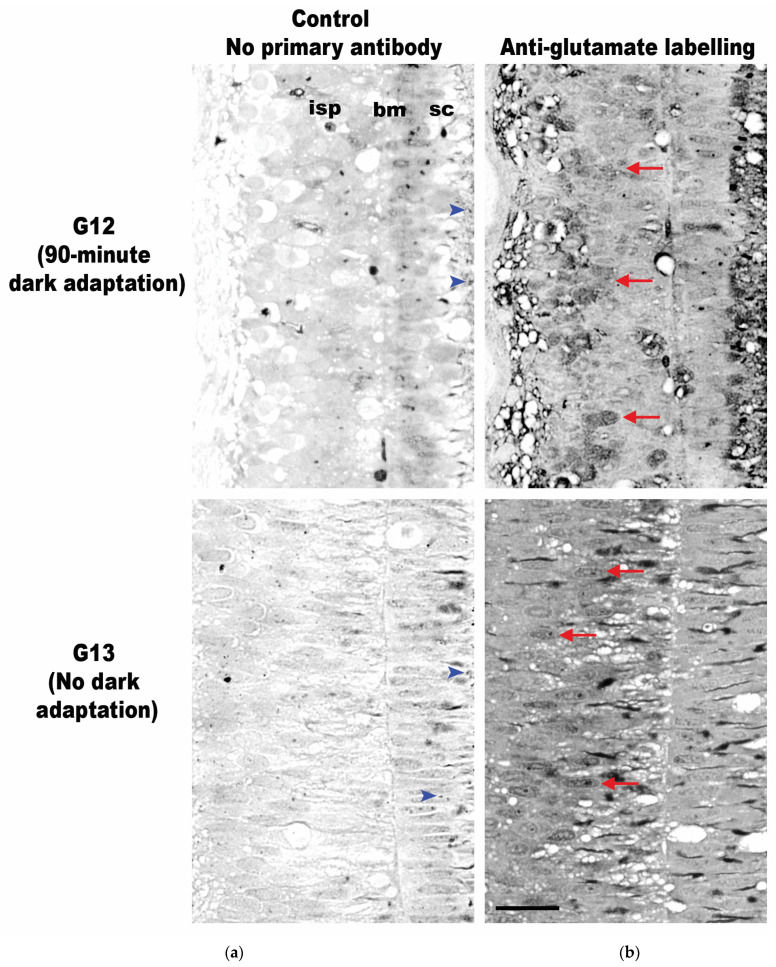
Transverse retinal cross-sections of the inner photoreceptor layer of juvenile *G. onyx* (G12–G13, both 15 mm ML). (**a**) Retinal sections without primary anti-glutamate serve as negative controls. (**b**) Retinal sections labeled with antibodies indicate the expression of glutamate neurotransmitters (dark grey areas). Glutamate expression in retinas subjected to darkness for 90 min (G12, top row) or without dark adaptation (G13, bottom row) following light exposure (350 lux for 30 min). Arrows indicate examples of cells reactive to the antibody. Arrowheads indicate ommin pigment granules within the supporting cells not destroyed by the preservation process. Abbreviations: supporting cells (sc), basal membrane (bm), inner layer of photoreceptors (isp). Scale bar = 20 µm.

The G12 and G13 retinas were labeled with an anti-glutamate antibody, and both specimens exhibited glutamate labeling within both the inner and outer segments of the photoreceptors (Figure 9). This technique involves the deposition of silver in cells in proportion to the quantity of antibody present, leading to the formation of various shades of grey in different areas. To ensure the specificity of the labeling, each tissue sample was processed with and without the anti-glutamate antibody. The ‘silver only’ negative controls in specimens G12 and G13 showed uniformly low and weak silver staining (top and bottom row, Figure 9a). However, the samples that were treated with the anti-glutamate antibody showed strong staining through the tissue and clearly identified cells were positive for glutamate labeling, with the highest concentration of glutamate appearing within the inner photoreceptor layer (top and bottom row, Figure 9b). The qualitative assessment of the two conditions appears to show that in the specimen allowed to adapt to darkness for 90 min (G12, top row, Figure 9b), glutamate labeling occurred in discrete cells; in comparison, the specimen with no subsequent dark adaptation period after light exposure (G13, bottom row, Figure 9b) exhibited similar labeling in all its inner photoreceptor cells.

## 4. Discussion

Recent studies have revealed an intriguing phenomenon in the migration dynamics of ommin pigment within cephalopod photoreceptors. While the migration of pigment to the distal tips is well documented, the return of pigment to the baseline is an area of growing interest. This return to the baseline may indicate a regulatory mechanism that maintains pigment distribution within the photoreceptor outer segments, ensuring optimal visual performance under varying light conditions. This study aimed to investigate the mechanisms underlying this pigment redistribution to improve our understanding of the adaptive strategies of cephalopod visual systems.

The results support the presence of migratory ommin pigment within the retinas of juvenile *Gonatus onyx*, suggesting that these animals can screen light in illuminated conditions. Unlike in coastal specimens, where dark adaptation results in the return of all pigment granules to the ommin layer, some pigment granules were clearly visible within the photoreceptors even after prolonged periods of darkness, providing the first experimental result of this mechanism and its potential time delay within a deep-sea oegopsid squid.

The use of ommochromes as a screening pigment has been reported in the retinas of major invertebrate groups including insects (*Drosophila melanogaster* [21]; *Deilephila elpenor* [22]; *Manduca sexta* [23]), crustaceans (*Procambarus clarkia* [24]; *Orconectes limosus* [25]), and chelicerates (*Limulus polyphemus* [9]), all of which are shallow-water or terrestrial species with pigments that migrate in coordination with the day/night cycle. Extensive studies, mostly on *D. melanogaster*, have shown that ommochromes are photosensitive colored compounds derived from tryptophan metabolism [26]. They are synthesized and stored in lysosome-related organelles called ommochromasomes, and are categorized into three groups, ommatin, ommin, and ommidin; for the latter two, little is understood of their chemistry and structure [27].

In juvenile *Gonatus* specimens exposed to light for 20 or 30 min, most pigment (but not all) returned to the basal layer under dark conditions, suggesting that an extended light response occurs, and may take more than 24 h to reverse. If the mechanisms controlling the return of the pigment granules to the photoreceptor bases are the same in *G. onyx* as in *Doryteuthis pealeii*, as described by Daw and Pearlman [3], then it is likely that the light adaptation conditions of 350 lux were strong enough to isomerize more than 10% of rhodopsin, which would elongate the time required for the pigment to fully return to its basal layer. Since *G. onyx* juveniles are typically distributed around 300–400 m deep [13], illumination at 350 lux is likely far brighter than any natural condition they would encounter. Furthermore, the spectral distribution of light emitted from fluorescent bulbs, such as those used in this study, typically has a spectral peak near 480–500 nm, aligning well with the spectral absorbance of most cephalopod photopigments, which may be linked to inducing pigment migration [28]. Therefore, exposure to fluorescent lighting with an illuminance of 350 lux and a spectral emission inclusive of the 480–500 nm range for around 20–30 min does appear sufficient to trigger light adaptation in *G. onyx*, but the restoration of full dark adaptation may take far longer than the duration of our trials. Additionally, dark adaptation may take significantly longer in deep-sea cephalopods exposed to abrupt bright light. It is also possible that the duration and intensity of light conditions in this study irreversibly damaged the retina, preventing a return to full dark adaptation. Alternatively, some ommin granules may consistently be found within the outer photoreceptor layer under all conditions. In some species of blowflies (*Lucilia* spp.), ommochromes help maintain the photoreceptor by transporting degraded rhabdomere membranes [27].

This study also provided novel evidence that glutamate is present within the retina of *G. onyx*. Glutamate does appear to decrease after a longer duration in the dark, which agrees with the findings of D’Aniello et al. [11]. They identified significant levels of neurotransmitters in the retina of cephalopods; glutamate was shown to decrease under dark conditions. However, D’Aniello et al. [11] examined adults of shallow-water species, with a relatively different eye structure, that were subjected to darkness for five days, making their results and the results of this study difficult to compare directly. Whether neurotransmitters used in cephalopod vision differ among lineages, among species occupying different environments with distinct light regimes, or between adults and juveniles remains to be seen.

The life history of *G. onyx* makes it an interesting study subject for light/dark adaptation. Females brood their eggs for six to nine months at depths of up to 2500 m, where they sometimes release their hatchlings, but have also been observed doing so near the surface [14,29,30]. Brooding at such depths under aphotic conditions may reduce predation risks for the female and her eggs/hatchlings, while extended post-spawning egg care may allow for relatively large and precocious young to hatch, capable of migrating vertically to nutrient-rich shallow water [31]. Retinal screening pigments could be advantageous as the young squid traverse a range of light conditions between the aphotic zone and the nutrient-rich euphotic zone, and across their subsequent exposure to regular day/night cycles.

As hatchlings mature into juveniles, they appear to settle into a regular diel cycle, distributed across a broad depth range during the day (100–800 m, peaking at 300–400 m) and a narrower range during the night (0–400 m, peaking at 200–300 m) [13]. Juvenile *G. onyx* have higher metabolic rates than their adult counterparts, which may influence their schooling behaviors and daily vertical migrations [13]. Their screening pigment may support visual acuity across the broad range of light conditions to which they may be exposed throughout the day and night and may protect the retina during ascents into shallower water during crepuscular hours.

At about 30–35 mm ML, juveniles begin to descend into greater depths. Their metabolisms slow to accommodate a less mobile, more solitary lifestyle, and they are most frequently observed at 300–1000 m (peaking at 700–800 m) during the day, and 100–800 m at night (peaking at 400–500 m) [13]. Thus, adult *G. onyx* may not experience the same diel range of light conditions, and screening pigment may play a less significant role. One exception may be females releasing hatchlings, which have been observed in near-surface waters [30]. It has been theorized that observations of deep-sea releases are the result of external stressors, such as net capture or predation, forcing the brooder to release her clutch prematurely, which has also been observed in octopods [29,32]. Females that release eggs in shallow water may benefit from screening pigment as they pass potential predators while migrating through brighter conditions. To determine whether ommin pigment migration occurs past the juvenile stage, more morphological investigations of adult *G. onyx* must be conducted.

The migration of these pigment granules in octopuses is controlled by melatonin circadian rhythms and varies during dark and light exposure [33]. Dopaminergic signals also act to modulate the basal migration of the granules, simulating dark adaptation conditions; efferent nerve cells release dopamine that acts on the retina and triggers the inward migration of the screening pigment, allowing maximum photoreceptor activity [6]. This study provides the first report of confirmed ommin pigment migration in an oegopsid squid. Evans et al. [8] reported an observation of apparent arrested screening pigment migration in the retinas of preserved juvenile *Teuthowenia pellucida*. This glass squid similarly inhabits well-lit shallow waters during its early life stages, then descends to greater depths as adults. Evans et al. [8] did not observe any ontogenetic variation in the thickness of the ommin layer, despite a disproportionate increase in photoreceptor length compared to the overall growth, and it remains unclear whether screening pigment migration remains possible and/or advantageous for deep-sea dwelling adult *T. pellucida*. However, the findings from the current study and Evans et al. [8] suggest that ommin migration does occur in at least some deep-sea squids across life stages that dwell primarily below the photic zone.

More research is required to fully understand the complex mechanisms governing screening pigment migration in the retina of deep-sea squids and the role neurotransmitters play. Future research on *G. onyx* screening pigment and its potential correspondence to dopamine levels should aim to determine a more precise time scale for light and dark adaptations in the retina and investigate a wider range of ontogenetic stages. Additionally, if interindividual variation exists, and if the mechanisms controlling ommin migration undergo ontogenetic modifications that are linked to visual transmission, those phenomena can start to be deciphered. More broadly, future research may aim to identify the variation in screening pigments among closely related species, and whether this mechanism is present in other oegopsids.

## Figures and Tables

**Table 1:** List of *G. onyx* identification numbers (ID) with corresponding mantle lengths (ML), light exposure and dark adaptation times for each specimen and their use in the analysis of pigment migration (PM) or neurotransmitter expression (NE). G02–G06 were grouped together for PM analysis (Group 1) and G07–G11 were in Group 2. G01 served as the negative control (NC) for PM analysis.

ID	ML (mm)	Analysis Conducted	Light Exposure (min)	Dark Adaptation (min)
G01	30	PM, NC	90	1440 (24 h)
G02	15	PM, Group 1	20	15
G03 *	15	PM, Group 1	20	30
G04	15	PM, Group 1	20	45
G05	15	PM, Group 1	20	60
G06	15	PM, Group 1	20	75
G07	10	PM, Group 2	30	20
G08	10	PM, Group 2	30	30
G09 *	20	PM, Group 2	30	40
G10	10	PM, Group 2	30	50
G11	10	PM, Group 2	30	60
G12	15	NE	30	90
G13	15	NE	30	0

* G03 and G09 were excluded from analysis.

## Data Availability

All data have been included in the manuscript.
